# Thalidomide-mediated immunomodulation in a patient with *IL-10RA* deficiency-associated very early-onset inflammatory bowel disease

**DOI:** 10.3389/fimmu.2026.1748151

**Published:** 2026-04-15

**Authors:** Aiyan Ren, Qian Zeng, Bin Zhang, Xue Chuan, Yalin Wu, Meiting Wang, Huan Ren, Yan Chen, Pei Huang, Zuochen Du

**Affiliations:** 1 Department of Pediatrics, Affiliated Hospital of Zunyi Medical University, Zunyi, China; 2 Guizhou Children’s Hospital, Zunyi, China

**Keywords:** IL-10RA, inflammatory bowel disease, mutation, T lymphocytes, thalidomide

## Abstract

**Introduction:**

Very early-onset inflammatory bowel disease (VEO-IBD) is frequently triggered by monogenic defects in the pathways responsible for immune system regulation, including mutations in the interleukin-10 receptor alpha (*IL-10RA*) gene. Patients often show severe and refractory clinical manifestations. Thalidomide has shown immunomodulatory effects in pediatric IBD; however, its immunological effects in *IL-10RA*-associated VEO-IBD remain poorly defined.

**Methods:**

A female patient aged 2 months diagnosed with VEO-IBD was enrolled in the study. Clinical data were gathered over time. Whole-exome sequencing was utilized to uncover pathogenic variants for genomic analysis. The expression of *IL-10RA* was assessed using Western blotting. Flow cytometry was performed both before and after treatment to evaluate the effect of the administration of thalidomide on the populations of T and B lymphocytes, alongside the immune activation levels.

**Results:**

The patient experienced chronic diarrhea, failure to thrive, and recurrent infections. Genetic analysis revealed the presence of compound heterozygous mutations in the *IL-10RA* gene (c.301C>T and c.537G>A). Western blotting analysis revealed a reduction in the levels of IL-10RA protein expression. Immunophenotyping showed an increase in the percentages of γδ T cells, Th17 cells, transitional B cells, and activated CD8^+^ T cells before treatment. After thalidomide therapy, the symptoms of diarrhea resolved, the growth parameters improved, the proportions of γδ T cells and transitional B cells decreased, and the inflammatory markers returned to normal levels.

**Discussion:**

This study confirms the immune dysregulation caused by *IL-10RA* gene mutation in VEO-IBD and demonstrates that thalidomide treatment can improve clinical symptoms and modulate specific lymphocyte subsets. The immune mechanisms behind the response to thalidomide in *IL-10RA*-associated VEO-IBD should be further explored.

## Introduction

Inflammatory bowel disease (IBD) encompasses a range of chronic inflammatory conditions affecting the gastrointestinal tract, and it is classified into three categories: IBD type unclassified (IBD-U), Crohn’s disease (CD), and ulcerative colitis (UC) (1). The underlying causes and disease mechanisms of IBD remain unclear (1, 2), and there is a rising trend in the number of children diagnosed with IBD (3). Very early-onset inflammatory bowel disease (VEO-IBD) is characterized by the appearance of symptoms prior to the age of 6 years and accounts for approximately 25% of all cases of IBD in pediatric patients (4). Individuals from this atypical group demonstrate a greater degree of severe disease features and a less favorable response to conventional treatment (4). In VEO-IBD, a genetic predisposition is much more relevant than in the case of adolescent or adult onset (5). According to genetic studies conducted in IBD, VEO-IBD is often associated with low-frequency monogenic variants (6). The susceptibility genes interleukin-10 (*IL-10*) and its receptor (*IL-10R*) are now widely recognized as key contributors to the pathogenesis of the disease (7, 8).

IL-10 is predominantly produced by dendritic cells, lymphocytes, and antigen-presenting cells (9). An essential component of the IL-10 signaling pathway is the interleukin-10 receptor alpha (IL-10RA), which serves as a subunit for the anti-inflammatory cytokine IL-10 (10). This signaling can regulate populations of immune cells, including macrophages, dendritic cells, and T cells, while also stopping the synthesis of pro-inflammatory cytokines such as tumor necrosis factor-α (TNF-α), interleukin-1β (IL-1β), and interleukin-6 (IL-6) (8, 9). Furthermore, it plays a role in modulating the differentiation of different lymphocyte subtypes, including T helper 1 (Th1), Th2, Th17, T follicular helper (Tfh) cells, and regulatory T cells (Tregs) (11–14). IL-10 can activate CD8^+^ T cells, enhancing IFN-γ secretion and cytolytic factors (15). In addition to assisting the survival of B cells, IL-10 also stimulates their proliferation and participates in the regulation of antibody production (13). Despite the IL-10/IL-10R signaling pathway being significant, there is limited understanding of how its defects affect the lymphocyte subgroups in VEO-IBD.

Impairments in the IL-10/IL-10R signaling pathway result in immune system imbalances and are correlated with more severe disease events, increased relapses, and poor response to treatment (7, 8). Biologic therapies are a promising treatment for refractory IBD. Thalidomide was introduced in adult patients with UC in 1979, and the drug subsequently became recognized as an important treatment for refractory IBD in adults and children (16–18). The immunomodulatory role of thalidomide may strike a balance between inhibiting the cytokine production from monocytes and macrophages while enhancing T-cell co-stimulation, leading to improved stimulation for T-cell proliferation and lymphokine release when combined with other activating signals (19, 20). Its principal mechanism involves binding to cereblon (CRBN), which modifies the substrate specificity of the CRBN–CRL4ACE3 ubiquitin ligase pathway, leading to the degradation of the transcription factors Ikaros (IKZF1) and Aiolos (IKZF3) (21). Within T cells, this degradation alleviates the inhibition of the *IL-2* gene, promotes interleukin-2 (IL-2) synthesis, and stimulates both T cells and natural killer (NK) cells (21). While this underlying mechanism is well understood, the immunomodulation effectiveness of thalidomide for VEO-IBD linked to *IL-10RA* mutations necessitates further exploration.

This study conducted a systematic clinical investigation of a pediatric patient with *IL-10RA* mutations and allowed for a longitudinal laboratory follow-up. Western blotting analysis was performed to measure the protein expression levels, and flow cytometry was conducted to analyze the changes within immune cells (T and B lymphocytes) and their activation, exhaustion profiles, and expression of the receptors of inflammation-related cytokines. Various immunological parameters were analyzed following thalidomide treatment in order to explore immunological correlates of the therapeutic response. The research aimed to provide insights into the mechanisms of immune dysregulation caused by impaired IL-10 signaling, to explore the immunomodulatory effect of thalidomide, and to offer a conceptual basis for individualized evaluation and targeted therapy for monogenic IBD.

## Materials and methods

### Patient

A 2-month-old female patient was recruited in this study. Relevant clinical information was collected after obtaining written informed consent from her parents. A control group comprised healthy children (healthy controls, HCs) of similar age who visited the hospital for regular health checkups. All procedures were conducted in accordance with the principles outlined in the Helsinki Declaration and obtained approval from the Ethics Committee at the Affiliated Hospital of Zunyi Medical University.

### Gene analysis

DNA was extracted from the peripheral blood mononuclear cells (PBMCs) of the patient, as well as from HCs, using the QIAamp DNA Mini Kit (Qiagen, Hilden, Germany) in accordance with the manufacturer’s instructions. Whole-exome sequencing (WES) was performed on the Illumina Genome Analyzer platform (MyGenostics, Beijing, China). To amplify the region of interest for the mutation, PCR was executed with the following primers: Exon3 F (5′-CCTGGTATCTCCTCAGGTATGGAA-3′), Exon3 R (5′-GGGACTTCAGAGCCATGTTCTAAG-3′), Exon4 F (5′-CATCCTCGGGAAGATTCAGCTACC-3′), and Exon4 R (5′-CCCAGACCCTCCCTTTAGTCCATA-3′). All primers were synthesized by Sangon Biotech (Shanghai, China). The PCR products were subsequently verified through Sanger sequencing (Sangon Biotech, Shanghai, China). The pathogenicity of sequence variants was evaluated according to the guidelines jointly issued by the American College of Medical Genetics and Genomics and the Association for Molecular Pathology (ACMG/AMP 2015).

### Flow cytometry

For experiments assessing the effects of the *IL-10RA* mutations and thalidomide treatment on immune function, 50 μl whole blood samples were collected and processed as previously described (22) (samples were collected from the patient at 2 months 14 days and at 5 months 17 days of age). Lymphocytes from the peripheral blood of the patient and age-matched HCs were labeled using the following antibodies: PE anti-human CD21 (354904), Brilliant Violet 421 anti-human IgM (314516), PerCP anti-human CD38 (303520), Brilliant Violet 510 anti-human IgD (348220), PE/Cy7 anti-human CD27 (302838), APC anti-human CD19 (302212), APC/Cy7 anti-human CD31 (303120), AF488 anti-human CD24 (311108), Brilliant Violet 711 anti-human IgG (410740), APC/Cy7 anti-human CD23 (338520), PerCP anti-human CD3 (300326), FITC anti-human CD4 (300506), APC anti-human CXCR3 (353708), Brilliant Violet 510 anti-human CD8a (301048), PE anti-human CD127 (351304), PE/Cy7 anti-human CD45RA (304126), Brilliant Violet 421 anti-human CD185 (356920), PB anti-human CD38 (356628), Brilliant Violet 605 anti-human CD27 (302830), PE/Fire 640 anti-human CD196 (353449), Brilliant Violet 711 anti-human CD45RO (304236), Brilliant Violet 785 anti-human PD-1 (329930), PE/Dazzle 594 anti-human CD57 (359620), AF660 anti-human TCRγ/δ (331240), Brilliant Violet 650 anti-human CD25 (302634), and APC/Fire 810 anti-human-HLA-DR (307674). All antibodies were purchased from BioLegend (San Diego, CA, USA) and stained according to the manufacturer’s instructions. All samples were analyzed on a Cytek® Aurora/Northern Lights™ (Cytek, DE, USA), and data were analyzed using FlowJo analysis software.

### Western blotting

The PBMCs derived from both the patient and the HCs were subjected to lysis using RIPA buffer (GB15618-100; ServiceBio, Wuhan, China), which contained enzyme inhibitors. The lysate was subsequently mixed with a loading buffer and denatured, then subjected to separation via 10% SDS-PAGE. After the separation process, the proteins were then transferred onto a PVDF membrane (Millipore, Darmstadt, Germany) and were subsequently blocked with a 5% milk solution. The membrane was then incubated with primary antibodies against anti-IL-10RA (13356-1-AP; Proteintech, Wuhan, China) and anti-GAPDH (T0004, Affinity, OH, USA). After this incubation period, a secondary antibody (Cell Signaling Technology, Danvers, MA, USA) was applied and the membrane washed with TBST. Finally, detection was performed utilizing a chemiluminescence detection system (Bio-Rad, Hercules, CA, USA).

### Multi-cytokine assay

Sera were obtained from the patient (at 2 months 14 days and at 5 months 17 days of age) and from age-matched HCs. Quantification of the cytokine levels was performed utilizing the LEGENDplex™ Multi-Analysis Flow Assay Kit (740808; BioLegend, San Diego, CA, USA), following the guidelines provided by the manufacturer. A FACS Canto Plus flow cytometer (BD Biosciences, San Jose, CA, USA) was utilized for analysis, and the collected data were processed using the LEGENDplex™ data analysis software platform.

### Summary of previously reported cases with *IL-10RA* c. C301T/G537A

We conducted a thorough review and integration of the existing literature on *IL-10RA* c.C301T/G537A mutations to clarify the clinical range of IBD.

### Statistical analysis

All experimental results presented herein are descriptive, and no statistical comparisons were conducted. All data visualizations were generated using GraphPad Prism 10.

## Results

### Clinical presentation

A female infant, the second child of non-consanguineous healthy parents, was born full term through cesarean delivery and had received routine vaccinations, showing normal development until the onset of symptoms. At 6 days of age, the patient developed recurrent diarrhea and fever, which remained poorly controlled despite monitoring. Her sibling did not experience any similar symptoms. By the time the patient was 1 month 12 days of age, she had developed anemia, persistent fever, abnormal liver function, bloody stools, and respiratory infection. The symptoms were present until the age of 2 months and 14 days, with chronic diarrhea being the most marked clinical feature throughout the entire course of the disease. During hospitalization, there was a significant fluctuation in the laboratory parameters. There was a significant increase in the liver enzymes. The peak levels of alanine aminotransferase (ALT) and aspartate aminotransferase (AST) reached as high as 643 and 351 U/L, respectively. The complete blood count showed that the patient was anemic, with a hemoglobin concentration of 86 g/L. The elevated creatine kinase-MB (CK-MB) level of 57 U/L suggested myocardial involvement, and urinary galactosamine testing showed a positive result. Testing for respiratory pathogens revealed the presence of methicillin-resistant *Staphylococcus aureus* (MRSA) and respiratory syncytial virus (RSV). The colonoscopy showed gastrointestinal tract damage, with linear and aphthous ulcers, Proctosigmoiditis, and anal fissure. The rectal biopsy findings showed chronic active inflammation. Microbiota analysis also revealed third-degree dysbiosis. The disease burden has resulted in failure to thrive. Analysis of the phosphorylated signal transducer and activator of transcription (pSTAT3) levels revealed that the pSTAT3 signaling response to IL-10 receptor stimulation by IL-10 was defective.

**Figure 1:**
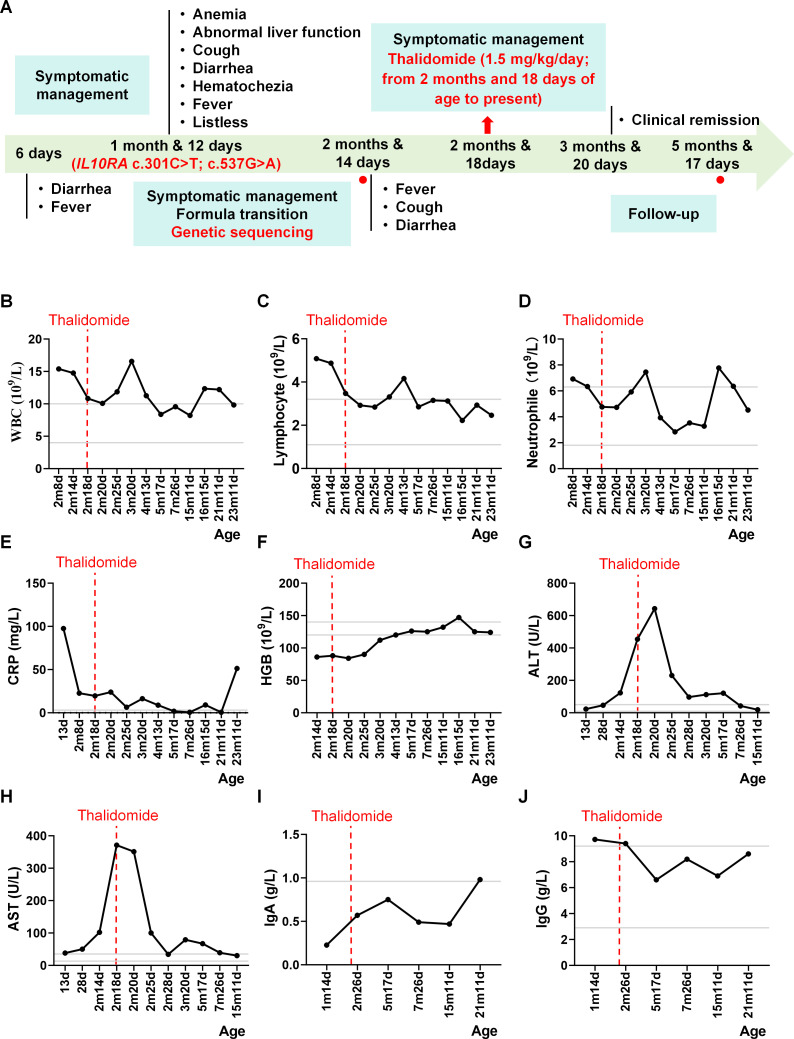
Clinical manifestations and laboratory findings of the patient. **(A)** Patient’s clinical manifestations and clinical course. *Red dots* represent the peripheral blood sampling time points. **(B)** White blood cell (*WBC*) counts. **(C)** Lymphocyte counts. **(D)** Neutrophil (*NEUT*) counts. **(E)** C-reactive protein (*CRP*) counts. **(F)** Hemoglobin (*HGB*) levels. **(G)** Alanine aminotransferase (*ALT*) levels. **(H)** Aspartate aminotransferase (*AST*) levels. **(I)** Immunoglobulin A (*IgA*) levels. **(J)** Immunoglobulin G (*IgG*) levels. *Gray lines* indicate the reference ranges.

The patient received antibiotics for pneumonia, an appropriate formula for better nutritional condition, and corticosteroids and intravenous immunoglobulins for the management of gastrointestinal symptoms and myocardial injury. The hematological parameters and febrile conditions improved after these series of interventions. Nevertheless, refractory diarrhea continued despite these measures. At the age of 2 months and 18 days, thalidomide (1.5 mg kg^−1^ day^−1^, continued to date) treatment was started, which was a turning point that controlled the diarrhea and ensured catch-up growth (**Figure 1A**). **Table 1** summarizes the key clinical parameters, laboratory abnormalities, microbiological findings, treatment details, and prognosis.

**Table 1:** Summary of the clinical manifestations of the patient.

System	Clinical feature	Laboratory result	Treatment	Outcome
Respiratory system	Recurrent pneumonia	Methicillin-resistant *Staphylococcus aureus* and respiratory syncytial virus	Antibiotic (intermittent use from 6 days to 2 months and 29 days of age)	Remission
Digestive system	Liver dysfunction	ALT: 19–643 U/L AST: 30–371 U/L	Supportive	Cure
	Recurrent diarrhea	Colonoscopy: linear and aphthous ulcers, proctosigmoiditis, and anal fissure	Supportive and replacement of the Neocate formula	Remission
Circulatory system	Myocardial injury	CK-MB: 57 U/L	Supportive	Cure
Immune system	high IgG	IgG: 6.6–9.72 g/L IgA: 0.227–0.75 g/L	IVIG	Remission
Hematologic system	Anemia	HGB: 86–147 g/L	NA	Remission

*NA*, not applicable; *AST*, aspartate aminotransferase; *ALT*, alanine aminotransferase; *CK-MB*, creatine kinase-MB; *IgG*, immunoglobulin G; *IgA*, immunoglobulin A; *HGB*, hemoglobin.

Repeated measurements of the inflammatory markers and liver enzymes showed normalization of the majority of the parameters following thalidomide treatment. The counts of white blood cells, lymphocytes, neutrophils, hemoglobin, and transaminases normalized (**Figures 1B–H**). The immunological indicators before and after immunotherapy exhibited decreased immunoglobulin G (IgG) levels, while the immunoglobulin A (IgA) levels were normal during the entire treatment (**Figures 1I, J**). The results of the laboratory tests observed thus far are consistent with the possibility that the patient’s refractory diarrhea may be due to immune dysregulation rather than classical gastrointestinal pathology, providing a mechanism by which thalidomide may be therapeutically effective in this case of *IL-10RA* deficiency.

### Identification of the *IL-10RA* mutations

**Figure 2:**
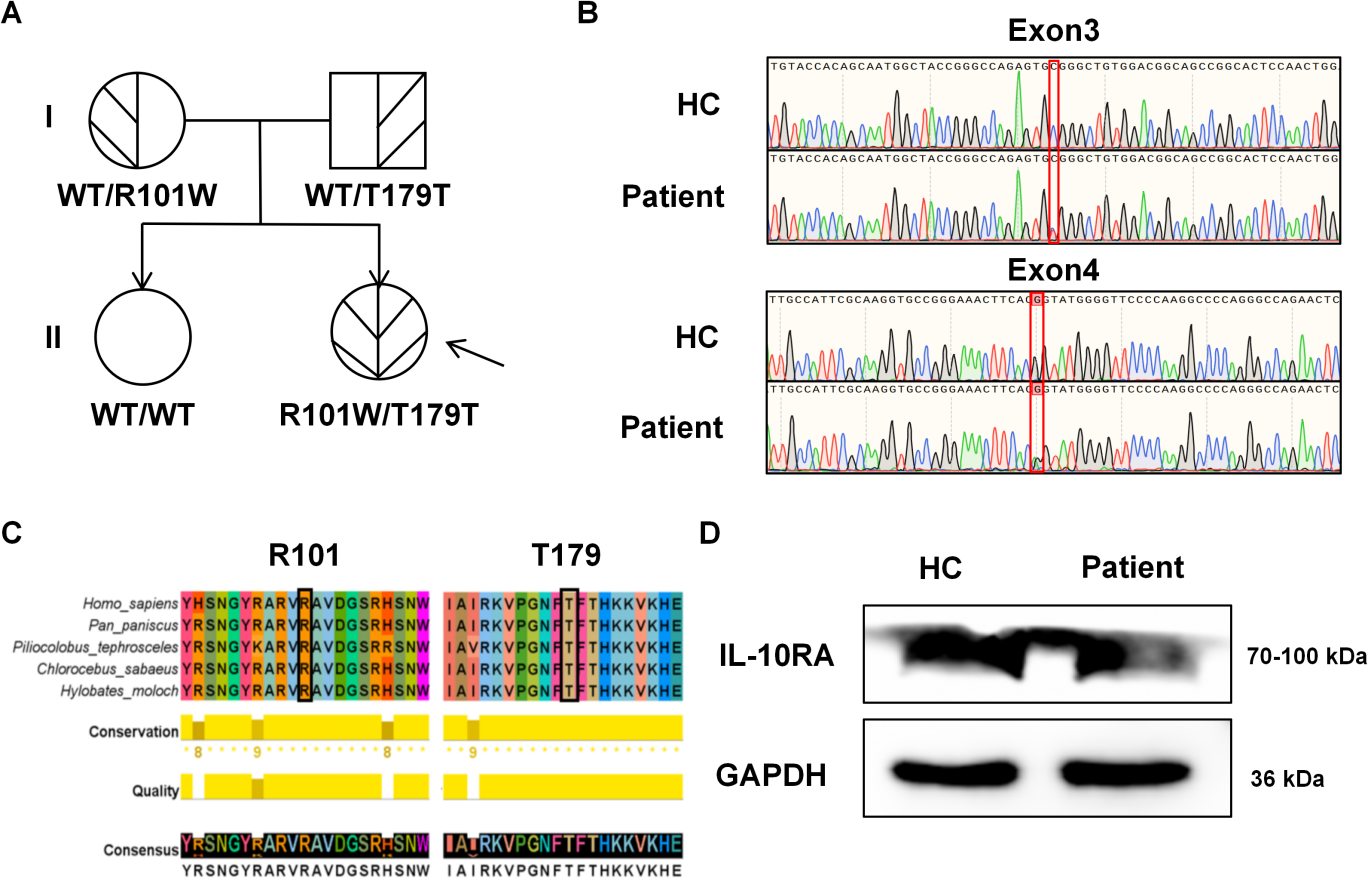
Identification of the *IL-10RA* p.R101W and p.T179T mutations. **(A)** Family pedigree: males are represented by *squares* and females by *circles*, with the *arrow* indicating the proband. **(B)** Sanger sequencing of *IL-10RA* in the patient and healthy controls (HCs). The c.301C>T; c.537G>A mutations were detected in the patient. **(C)** Multiple sequence alignment showing conservation of the exon 3 and exon 4 domains across species. **(D)** Western blot analysis revealed that the patient exhibited reduced *IL-10RA* expression levels compared with age-matched HCs.

The patient had refractory diarrhea, repeated infections, and severe failure to thrive, which did not respond to many common treatments. The colonoscopy findings revealed chronic inflammation and ulcerations of the intestine, which led to a diagnosis of VEO-IBD. WES identified a compound heterozygous mutation in the *IL-10RA* gene—c.301C>T (p.R101W; classification: pathogenic; ACMG criterion: PM3, PS3, PM2) and c.537G>A (p.T179T; classification: pathogenic; ACMG criterion: PM2, PM3, PP3)—in the patient. The mother carried the p.R101W variant and the father carried the p.T179T variant, which they passed on. The patient’s sister, who did not display any symptoms, did not have similar mutations (**Figure 2A**). Sanger sequencing confirmed the compound heterozygous genotype in the patient (**Figure 2B**). The alignment of protein sequences from different species showed that these two residues are highly conserved (**Figure 2C**) and that their modification may disrupt the function of IL-10RA. The autosomal-recessive nature of the defects in IL-10/IL-10R signaling indicated that homozygous and compound heterozygous mutations are responsible for monogenic IBD (23).

Western blotting analysis was conducted on the PBMCs from the patient and age-matched HCs to evaluate the potential impact of the identified mutations on protein expression. The results showed that the patient group had lower levels of IL-10RA protein than the controls (**Figure 2D**). This reduction was consistent with impaired anti-inflammatory function of the IL-10 signaling pathway.

### Abnormal changes in the T-lymphocyte subsets of the patient

Earlier studies indicated that IL-10 is critical for immune response regulation in the intestines of mice (24). To understand how IL-10 signaling disruption affects immune cells, we analyzed the lymphocyte subsets in this patient. Our findings indicate that, in comparison to age-matched HCs, there were no significant alterations in the overall counts of T cells in the patient. Nevertheless, there was a notable reduction in the levels of CD4^+^ T cells, accompanied by a diminished CD4/CD8 ratio (**Table 2**).

**Table 2:** Analysis of the patient’s immune cell subsets before and after thalidomide treatment.

Lymphocytes	2 months and 14 days (before thalidomide treatment)				5 months and 17 days (after thalidomide treatment)			
	Percentage	Reference range	Number (µl)	Reference range	Percentage	Reference range	Number (µl)	Reference range
T cells	64.3 (L%)	57.45–75.22	3,131.41	2,766.42–4,067.59	46.5 (L%)	57.45–75.22	1,325.25	2,766.42–4,067.59
CD8^+^ T cells	13.1 (L%)	12.61–25.08	637.97	657.97–1275.72	11.6 (L%)	12.61–25.08	330.6	657.97–1,275.72
CD8^+^ naive	86.9 (CD8%)	63.39–94.29	555.18	484.03–1,009.23	69.9 (CD8%)	63.39–94.29	231.42	484.03–1,009.23
CD8^+^ TEMRA	0.94 (CD8%)	0.00–10.48	5.84	0.00–109.15	2.05 (CD8%)	0.00–10.48	6.84	0.00–109.15
CD8^+^ CM	8.04 (CD8%)	5.51–27.25	51.62	45.95–255.50	16.4 (CD8%)	5.51–27.25	54.15	45.95–255.50
CD8^+^ EM	4.14 (CD8%)	0.02–4.74	26.30	0.24–52.35	11.7 (CD8%)	0.02–4.74	38.76	0.24–52.35
CD4^+^ T cells	17.1 (L%)	37.71–56.05	832.77	1,890.34–2,987.99	17 (L%)	37.71–56.05	484.50	1,890.34–2,987.99
CD4^+^ naive	77.8 (CD4%)	67.52–89.73	647.71	1,433.25–2,546.14	60.4 (CD4%)	67.52–89.73	299.25	1,433.25–2,546.14
CD4^+^ TEMRA	0.45 (CD4%)	0.00–0.68	3.75	0.00–16.32	0.47 (CD4%)	0.00–0.68	2.34	0.00–16.32
CD4^+^ CM	15.5 (CD4%)	9.21–32.18	129.06	239.11–676.23	27.3 (CD4%)	9.21–32.18	135.66	239.11–676.23
CD4^+^ EM	6.28 (CD4%)	0.16–1.82	52.11	3.46–42.05	11.7 (CD4%)	0.16–1.82	58.43	3.46–42.05
TCRαβ^+^ DNT	NA	NA	NA	NA	NA	NA	NA	NA
γδ T	4.47 (T%)	3.49–8.29	140.26	94.44–300.91	2.44 (T%)	3.49–8.29	32.21	94.44–300.91
CD4^+^/CD8^+^	1.31	1.62–3.77	NA	NA	1.47	1.62–3.77	NA	NA
B cells	8.89 (L%)	14.71–31.04	432.94	667.15–2,044.69	21.1 (L%)	14.71–31.04	601.35	667.15–2,044.69
UMB	0.36 (B%)	0.93–4.23	17.53	10.18–44.00	0.2 (B%)	0.93–4.23	1.14	10.18–44.00
Naive B	90.7 (B%)	87.99–94.63	392.5	597.44–1,940.71	95.4 (B%)	87.99–94.63	572.85	597.44–1,940.71
Transitional B	32.7 (B%)	11.77–30.45	132.5	122.42–444.63	19.5 (B%)	11.77–30.45	115.4	122.42–444.63
Plasmablast B	0.16 (B%)	0.69–3.42	0.6	8.35–36.25	0.18 (B%)	0.69–3.42	1.08	8.35–36.25

NA: Not Applicable. L%: percentage of lymphocytes; CD8%: percentage of CD8^+^ T cells; CD4%: percentage of CD4^+^ T cells; T%: percentage of CD3^+^T cells; B%: percentage of CD19^+^ B cells. CD8^+^ Naive (cytotoxic T lymphocyte with differentiation markers): CD3^+^CD8^+^CD45RA^+^CD27^+^; CD8^+^ TEMRA (terminally differentiated effector memory): CD3^+^CD8^+^CD45RA^+^CD27^-^; CD8^+^ CM (central memory): CD3^+^CD8^+^ CD45RA^-^CD27^+^; CD8^+^ EM (effector memory): CD3^+^CD8^+^CD45RA^-^CD27^-^; CD4^+^ Naive (helper T lymphocyte markers): CD3^+^CD4^+^ CD45RA^+^CD27^+^; CD4^+^ TEMRA (terminal effector memory differentiation): CD3^+^CD4^+^CD45RA^+^CD27^-^; CD4^+^ CM (central memory): CD3^+^CD4^+^CD45RA^-^CD27^+^; CD4^+^ EM (effector memory): CD3^+^CD4^+^CD45RA^-^CD27^-^; TCRaβ^+^ DNT: double negative T lymphocytes: CD3^+^TCRaβ^+^CD4^-^CD8^-^; Unswitched Memory B (UMB) cells: CD19^+^CD27^+^IgD^+^; Naive B cells: CD19^+^CD27^-^IgD^+^; Transitional B cells: CD19^+^CD24^++^CD38^++^; Plasmablasts B: CD19^+^CD24^-^CD38^++^.

**Figure 3:**
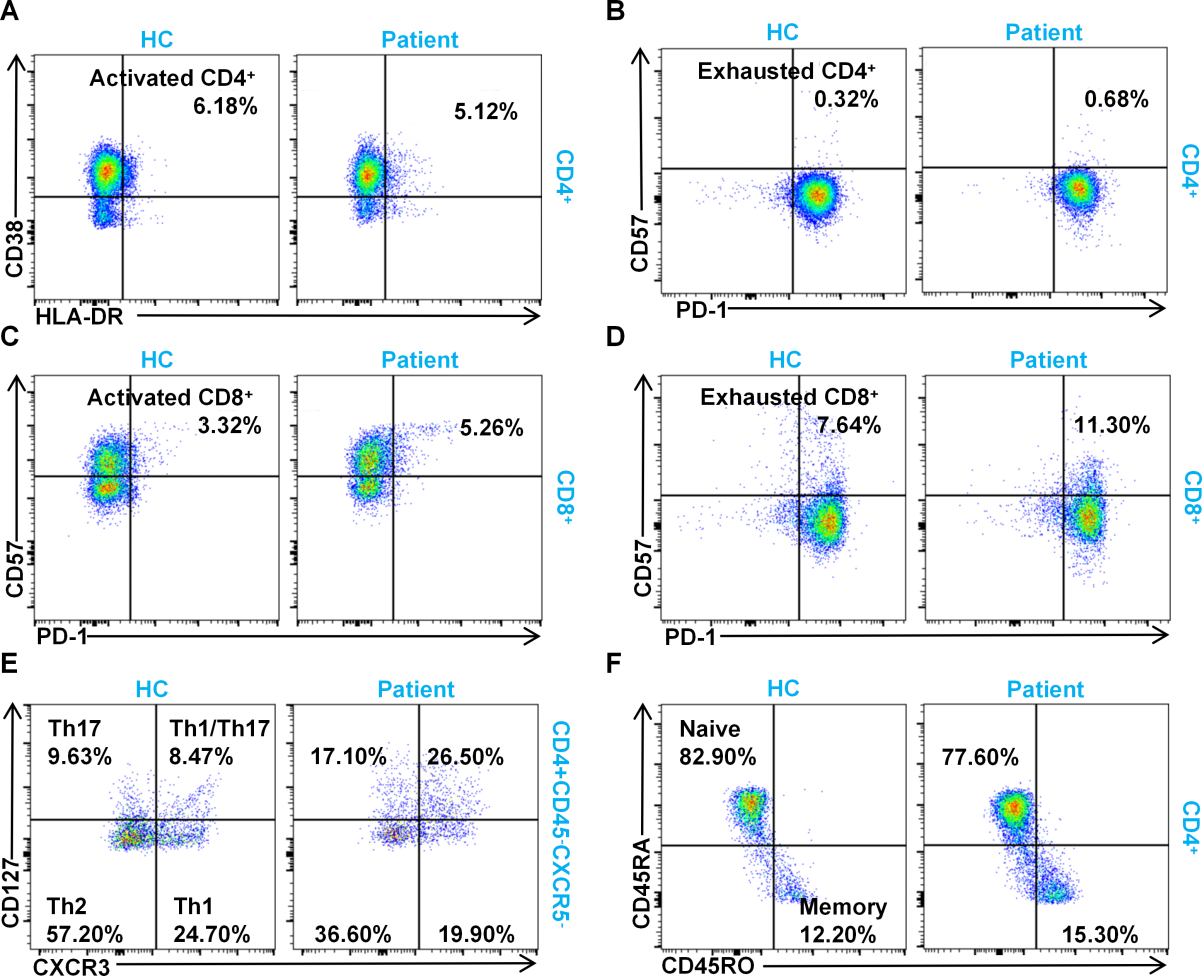
Alterations of the T-Lymphocyte subsets in the patient. Flow cytometry analysis of activated CD4^+^ T cells **(A)**; exhausted CD4^+^ T cells **(B)**; activated CD8^+^ T cells **(C)**; exhausted CD8^+^ T cells **(D)**; Th1, Th2, and Th17 subsets **(E)**; and naive and memory T cells **(F)**. Comparisons were made between the patient and three age-matched healthy controls (HCs). Representative flow cytometry plots are shown.

To characterize the immune dysregulation in this patient in detail, we set out to investigate abnormalities in the immune cells induced by the IL-10/IL-10R signaling dysfunction. We carried out extensive analyses of the T-cell subsets given that the dysregulation of this pathway causes aberrant proliferation of Th17 and Th17/Th1 cells (25). The results illustrated that CD4^+^ T cells were phenotypically switched from an activated to an exhausted state (**Figures 3A, B**). However, CD8^+^ T cells showed enhanced activation with gradual exhaustion (**Figures 3C, D**). As expected, there was an increase in the levels of Th17 and Th17/Th1 cells (**Figure 3E**). T-cell activation was accompanied by a rise in memory T cells and a drop in naive T cells (**Figure 3F**). These findings collectively indicate chronic T-cell activation and a systemic immune dysregulation state in the patient.

Consistent with these findings, our initial immunophenotypic analysis revealed a decreased total B lymphocyte count. Analysis of the subsets showed a lower frequency of plasmablast B and a higher percentage of transitional B cells than those in the age-matched HCs (**Table 2**). In contrast, the frequencies of the other B-cell subsets did not exhibit any significant differences between the patient and the control group.

### Changes in the immune function of the patient after thalidomide treatment

**Figure 4:**
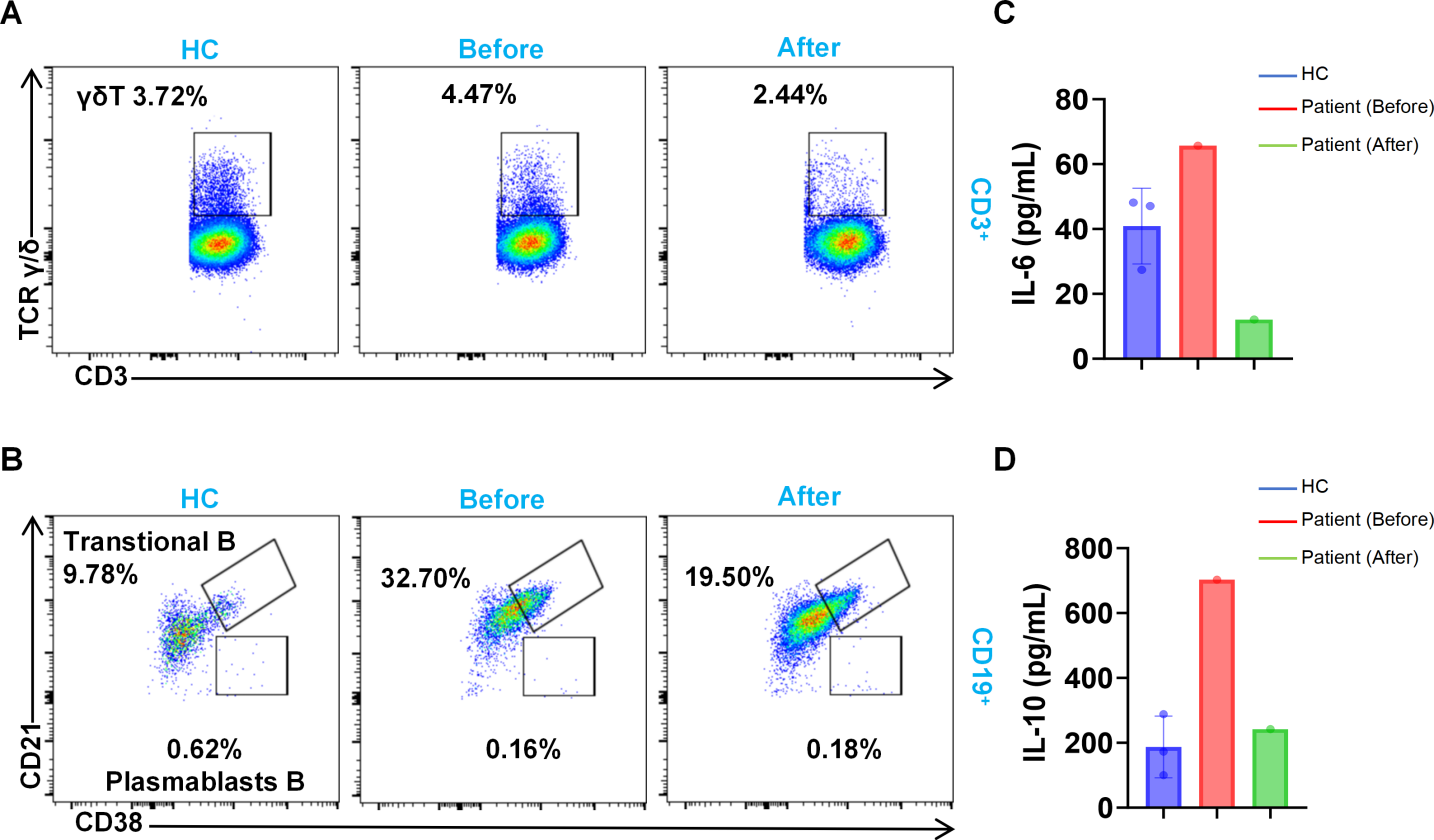
Changes in the immune function of the patient after thalidomide treatment. **(A, B)** Flow cytometry analysis of γδ T cells **(A)** and transitional B cells **(B)**. Comparisons were made between the patient and three age-matched healthy controls (HCs). Representative flow cytometry plots are shown. **(C, D)** Cytokine detection assays showing the expression of IL-6 **(C)** and IL-10 **(D)**.

It has been reported that thalidomide can alter CD4^+^ T-cell subset populations by downregulating effector T (Teff) cells while preserving Tregs (26). To shed light on the immunological changes in the patient, alterations in the lymphocyte subsets before and after thalidomide treatment were examined using flow cytometry. After thalidomide treatment, the overall amount of T lymphocytes was found to be less than the baseline levels. The counts of both CD4^+^ and CD8^+^ T cells were lower, with a drop mostly occurring within the CD4^+^ naive T-cell subset (**Table 2**). In addition, a substantial reduction in the γδ T-cell levels after treatment was identified (**Figure 4A**). Furthermore, transitional B cells (**Figure 4B**) showed a quantitative decrease among the B-lymphocyte subsets compared with those at pretreatment. These results indicate that thalidomide regulates multiple lymphocyte lineages, offering a cellular-level explanation of the immunomodulatory activity of thalidomide under *IL-10R* deficiency.

The immunomodulatory mechanisms of thalidomide, including its modulation of the IL-6 and IL-1β cytokines (27), have already been established. Multiplex cytokine analysis revealed that elevated serum levels of IL-6 and IL-10 are associated with *IL-10RA* mutations. After the administration of thalidomide, the concentrations of IL-6 (**Figure 4C**) and IL-10 (**Figure 4D**) returned to normal levels. Laboratory evaluations further confirmed that the patient’s TNF-α levels gradually decreased to a normal range (from 2.85 to 0.23 pg/ml) following treatment.

## Discussion

The present study describes the case of a 2-month-old female infant with recurrent diarrhea, infection, and failure to thrive. Laboratory tests showed an increase in the inflammatory indicators. Colonoscopy revealed gastrointestinal mucosal injury. WES identified compound heterozygous mutations in the *IL-10RA* gene. These genetic alterations resulted in reduced IL-10RA protein expression, thereby disrupting the anti-inflammatory function of IL-10 signaling. This study further confirms that *IL-10RA* mutations lead to immune dysregulation in VEO-IBD and demonstrates that thalidomide treatment alleviated the clinical symptoms and modulated specific lymphocyte subsets.

*IL-10RA* mutation is a pathogenic factor of VEO-IBD (28). The common hotspot mutation *IL-10RA* c.537G>A (p.T179=) is frequently accompanied by c.301C>T (p.R101W) in VEO-IBD (23). This synonymous mutation, often referred to as p.T179T, represents a distinct pathogenic variant with clinical significance (29). Although p.T179T does not affect the encoded amino acid, it alters normal mRNA splicing, resulting in abnormal transcript processing, which leads to the production of truncated, non-functional proteins that lack IL-10 signaling capacity (30). Overall, all previously reported cases harboring the *IL-10RA* c.301C>T/G537A mutation exhibited extremely early onset and severe and refractory phenotypes, often accompanied by extraintestinal manifestations, recurrent fever, cutaneous lesions, infections, growth retardation, and hematological abnormalities (**Table 3**). Consistent with these reported cases, although this patient presented with early-onset severe disease, no significant cutaneous and perianal findings were evident. For patients with homozygous or compound heterozygous mutations in *IL-10RA*, including p.T179T, early genetic testing is clinically impactful. Identification of these mutations is important for timely interventions and represents a prerequisite for achieving clinical remission. This underscores the importance of molecular diagnosis in the management of VEO-IBD.

**Table 3:** Summary of previously reported cases with *IL-10RA* c.C301T/G537A.

Gene	Mutant	Protein	Extraintestinal manifestation	Onset	Immunosuppression therapy	Reference
*IL10RA*	G537A	T179T	NA	Within 1 month	Steroids	(37)
*IL10RA*	G537A	T179T	Folliculitis	Within 1 month	ADA	(37)
*IL10RA*	C301T/G537A	R101W/T179T	Growth failure, persistent anemia, hypoalbuminemia	2 months	CS, AZA, IFX	(30)
*IL10RA*	C301T	Arg101Trp	Folliculitis	7 days	AZA, CS	(38)
*IL10RA*	C301T/G537A	R101W/T179T	Recurrent febrile, oral ulcer	Within 10 months	IFX, AZA, ADA	(30)
*IL10RA*	C301T/G537A	R101W/T179T	Rash, oral ulcers, purulent otitis media	Within 1 month	NA	(39)
*IL10RA*	G537A	T179T	High fever	6 years	AZA, IFX, ADA	(30)
*IL10RA*	G537A	T179T	Folliculitis	6 months	CS, IFX, ADA	(29)
*IL10RA*	G537A	T179T	Recurrent fever, failure to thrive	Within 1 month	NA	(40)
*IL10RA*	G537A	T179T	Perioral dermatitis	6 months	NA	(41)
*IL10RA*	G537A	T179T	Severe infection, severe anemia	2 months	Cyclosporine, steroids, ADA, IFX	(42)
*IL10RA*	C301T/G537A	R101W/T179T	Severe infection, anemia, abnormal liver function	6 days	CS, thalidomide	This study

*NA*, not applicable; *ADA*, adalimumab; *AZA*, azathioprine; *CS*, corticosteroids; *IFX*, infliximab.

Studies have shown the expansion of Th17 cells in IBD (31). The reported changes are consistent with the changes in the T-lymphocyte compartment of the patient in this study. Moreover, this individual exhibited a higher frequency of γδ T cells and transitional B cells. The administration of thalidomide led to the restoration of immune homeostasis, particularly by normalizing the response pathways of both transitional B cells and γδ T cells. These observations reinforce the idea that thalidomide has the potential to restore equilibrium in the disrupted immune network of patients suffering from VEO-IBD.

Thalidomide is a reasonable option for children and adolescents with refractory IBD despite considerable inter-individual variability in the treatment responses. Our data further support the clinical efficacy of thalidomide in VEO-IBD by highlighting its unique immunoregulatory effects on specific lymphocyte subsets. Thalidomide reduced the levels of transitional B cells and γδ T cells, which are associated with functional IL-10RA deficiency. γδ T cells and transitional B cells may serve as useful prognostic markers of treatment response and as essential mediators of cellular functional behavior in the immunopathogenesis of VEO-IBD. The reduction of these subsets after treatment may be mediated by the NF-κB signaling pathway through which B cells and T cells undergo functional and differentiation changes (32). In future research, the incorporation of single-cell sequencing technologies will enhance the investigation of the effects of thalidomide on the developmental trajectory of T and B cells in IBD and facilitate the identification of novel targets for precision immunotherapy.

The treatment of IBD now routinely involves immunomodulatory therapy (33). Nonetheless, studies specifically evaluating thalidomide in VEO-IBD remain limited. According to existing studies, thalidomide achieves a higher remission rate than other therapies in patients with severe VEO-IBD. However, some patients discontinue treatment due to suboptimal efficacy, and adverse reactions may also occur in a subset of individuals (17). The harmful effects of thalidomide limit its use (34). The common side effects include dizziness, somnolence, facial or limb edema, and gastrointestinal disturbances (35). More serious adverse effects, such as peripheral neuropathy, deep vein thrombosis, have also been reported (36). The subject has not yet exhibited any impairments; however, long-term monitoring and close observation are warranted.

As an inhibitor of TNF-α, thalidomide downregulates the inflammatory cytokines in children, including IL-1β and IL-6, and is associated with clinical improvement (27). In this case, thalidomide similarly decreased the level of IL-6. However, given the single-case nature of this study, the results should be interpreted with caution. In future research studies, larger patient cohorts with genetically confirmed *IL-10RA* mutations, together with more extensive immune profiling, will be required. The underlying signaling mechanisms can be further explored using gene-edited mouse models. In parallel, viral vector-based delivery of genome editing tools or *IL-10RA* variant constructs may enable cell-level dissection of specific mutation sites, allowing precise functional comparisons between wild-type and mutant *IL-10RA*. In addition, comparative analyses of patients harboring distinct disease-causing *IL-10RA* mutations may help elucidate the mechanisms underlying dysregulation of γδ T cells and transitional B cells. Such multidimensional research will deepen our comprehension of how *IL-10RA* mutations disrupt immune homeostasis and will ultimately guide the development of targeted therapeutic strategies for this genetically defined VEO-IBD.

## Conclusion

In conclusion, we report the extensive immunological phenotype of a VEO-IBD patient with rare compound heterozygous *IL-10RA* mutations (p.R101W and p.T179T). Our immune reconstitution analysis after thalidomide treatment showed transitional B cells and γδ T lymphocytes as the most responsive subsets. The immunomodulatory effects of thalidomide revealed by these findings require further investigation, particularly with respect to its impact on the T- and B-cell developmental pathways in the context of IBD pathogenesis. A thorough immunophenotypic characterization within this disease category has potential value for the evaluation of immune function and disease severity and may inform personalized therapeutic strategies. Changes in the lymphocyte subset counts may also reflect the status of immune recovery during remission periods. These data indicate that thalidomide deserves further investigation in *IL-10RA*-associated VEO-IBD in appropriately powered studies with rigorous statistical design. Such studies will help clarify both the mechanisms and the clinical role of thalidomide in this setting.

## Data Availability

The original contributions presented in the study are publicly available. This data can be found here: Genome Sequence Archive for Human (Accession: HRA017628).
